# Fatality Following Intentional Ingestion of *Cerbera odollam* Seeds

**DOI:** 10.5811/cpcem.2018.5.38345

**Published:** 2018-06-12

**Authors:** Ryan Misek, Glenn Allen, Valerie LeComte, Nicholas Mazur

**Affiliations:** * Midwestern University, Chicago College of Osteopathic Medicine, Department of Emergency Medicine, Downers Grove, Illinois; † Franciscan Health Hammond, Emergency Department Pharmacy, Hammond, Indiana

## Abstract

Seeds from the mangrove plant *Cerbera (C.) odollam*, known as the “suicide tree,” are responsible for a significant number of plant deaths worldwide but are not well recognized in Western medicine. Cerberin is a cardiac glycoside concentrated in the plant’s seeds, which causes disrupted cardiac electrical activity leading to fatal dysrhythmias. We present a fatal case of intentional *C. odollam* seed ingestion. The patient experienced high-degree heart block and cardiac arrest despite supportive treatment and digoxin immune fab administration. Clinicians should be aware of the potential morbidity and mortality associated with *C. odollam* poisoning and be prepared for resuscitative interventions.

## INTRODUCTION

Toxins found in the seeds of the tree *Cerbera (C.) odollam*, also known as *C. mangha*, are responsible for hundreds of deaths worldwide, with 537 recorded deaths from 1989 to 1999 in the southern Indian state of Kerala alone.^1^ *C. odollam* poisoning, however, has received little attention in Western medicine. Cerberin, a cardiac glycoside found in the seed, reversibly inhibits the sodium–potassium adenosine triphosphatase (Na–K– ATPase) exchanger in myocardial cells causing disruption in cardiac electrical activity and ultimately death.^1^,^2^ Cerberin is chemically related to other cardiac glycosides found in plant species including oleander (*Nerium oleander*), foxglove (*Digitalis lanata, Digitalis purpurea*), ouabain (*Strophanthus gratus*), and yellow oleander (*Thevetia peruviana*), which all produce similar effects as digoxin on the heart.^1^ *C. odollam* is referred to as the “suicide tree” or “pong-pong” tree, as its seeds continue to contribute to a significant number of suicides and homicides each year, particularly in rural areas of South Asia. We present a fatal case of intentional self-ingestion of “pong-pong” seeds in an adult patient who purchased the seeds online.

## CASE REPORT

A 22-year-old previously healthy, pre-operative male-to-female transgender patient presented via ambulance after a suicide attempt. The patient admitted to ingesting one “pong-pong” seed approximately seven hours prior to arrival and said that he had purchased the seed online after researching suicide. He admitted to vomiting about two hours after ingestion of the ground seed, and upon initial emergency department (ED) presentation, reported chest pain and “feeling weird.”

Past medical history included a history of depression, post-traumatic stress disorder, and one previous suicide attempt with inpatient psychiatric admission. ED vital signs revealed a temperature of 36.2° Celsius, pulse rate of 53 beats per minute (bpm), respiratory rate of 16 breaths per minute, blood pressure 114/54 millimeters of mercury, and pulse oximetry of 99% on room air. On physical examination, the patient was alert and oriented to person, place and time with a Glasgow Coma Scale of 15. Patient’s pupils were three millimeters and reactive bilaterally; moist mucous membranes were noted and cardiovascular examination revealed bradycardia with a regular rhythm and no murmurs. Lungs were clear bilaterally and the abdomen was soft without tenderness. Skin was warm and dry. Neurological examination revealed normal strength in all extremities without sensory deficits; no clonus or rigidity was noted, and reflexes were 2+ throughout.

**Image 1:**
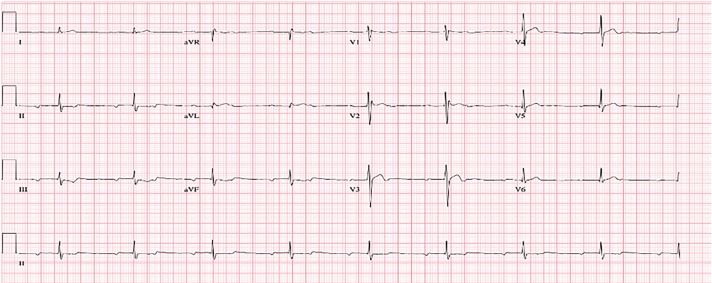
Initial electrocardiogram demonstrating second-degree heart block with 2:1 atrioventricular conduction and ST-segment depression with biphasic T-waves.

**Image 2:**
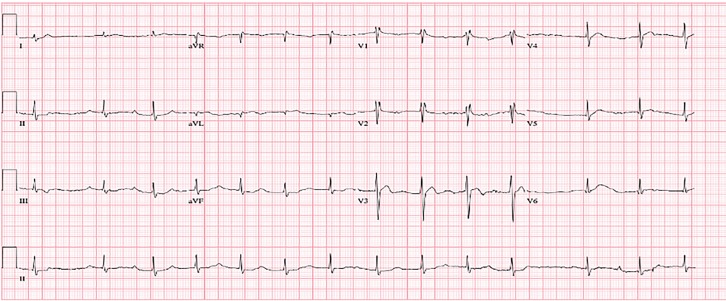
Electrocardiogram following initial dose of digoxin immune fab demonstrating sinus rhythm with first-degree atrioventricular block, persistent ST-segment depression with biphasic T-waves.

We notified Poison Control immediately upon the patient’s arrival, and initial recommendations were to give five vials of digoxin immune fab with hourly potassium levels and supportive care. Poison Control cautioned the patient could develop second- and third-degree heart block, dysrhythmias, and refractory hypotension. The patient’s initial electrocardiogram (ECG) (Image 1) demonstrated second-degree heart block with 2:1 atrioventricular (AV) conduction and ST-segment depression with biphasic T-waves. Five vials of digoxin immune fab were given intravenously and the patient’s heart rate improved to 90 bpm. The second ECG (Image 2) was obtained after the initial dose of digoxin immune fab and showed improvement to a sinus rhythm with first-degree AV block with persistent ST-segment depression with biphasic T-waves. Initial serum chemistries were significant for a potassium level of 5.2 milliequivalents per liter (mEq/L) (normal 3.5–5.1) and troponin I <0.05 nanograms per milliliter (ng/ml). Serum toxicology was negative for digoxin (1.3 ng/ml), ethanol (<10 milligrams per deciliter), acetaminophen (<10 micrograms per milliliter) and salicylates (<2.5 milligrams per deciliter); a urine drug screening was pending.

**CPC-EM Capsule**

What do we already know about this clinical entity?

Cerbera (C.) odollam seeds contain active cardiac glycosides and account for significant worldwide mortality. Management of C. odollam poisoning is similar to that of digoxin toxicity.

What makes this presentation of disease reportable?

We present the second known C. odollam poisoning in the United States and the first fatality reported, despite treatment with digoxin immune fab administration.

What is the major learning point?

Severe C. odollam poisoning is associated with hyperkalemia, bradycardia, and lack of response to atropine. Clinicians should prepare for aggressive resuscitative interventions.

How might this improve emergency medicine practice?

We hope to educate clinicians about the lethality and treatments of C. odollam poisoning, while raising awareness about a common international means of suicide.

**Image 3:**
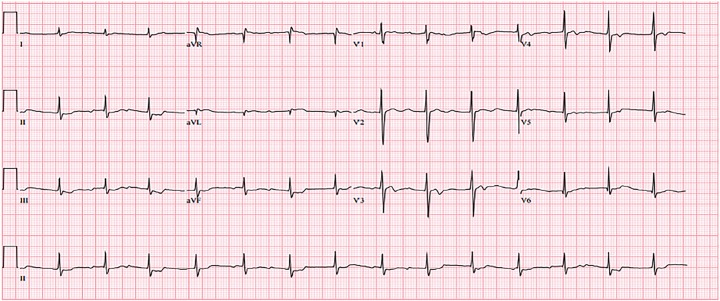
Third electrocardiogram after patient decompensation demonstrating high-degree heart block.

Approximately two hours into the ED course, the patient became bradycardic again. When a third ECG (Image 3) demonstrated a high-degree heart block, five more vials of digoxin immune fab were ordered. Repeat serum potassium level was 5.7 mEq/L. Thirty minutes later, the patient became unresponsive and lost pulses, and the monitor showed pulseless electrical activity as the repeat dose of digoxin immune fab was administered. The patient was resuscitated per Advanced Cardiovascular Life Support protocols. During resuscitation, he was intubated and a right femoral central venous catheter was placed. Ten additional vials of digoxin immune fab and lipid emulsion 20% (100 ml) were given intravenously. Ultimately, after two hours of unsuccessful resuscitation, the patient expired. Postmortem, forensic toxicology testing failed to identify a causative agent; however, a *C. odollam* test was sent to the Federal Bureau of Investigation laboratory in Quantico, Virginia, and was still outstanding at time of this publication.

## DISCUSSION

The *C. odollam* plant belongs to the Apocynacea family and grows predominantly in Southern India, Vietnam, Cambodia, Sri Lanka, Myanmar, Malaysia and Madagascar.^1^ Its seed is where the active cardiac glycosides cerberin, cerberoside, and odollin are concentrated.^1^, ^2^ Three glycosides are thought to be responsible for the toxicity of *C. odollam*: cerberin, cereberoside and odollin,^1^,^3^ while a fourth glycoside was identified in 2004.^4^ These toxic compounds are concentrated in the seeds and oil of the plant. An alcohol extract produced from seeds of *C. odollam* was shown to have similar effects on anesthetized dogs and cats as a digoxin solution, showing a rise in blood pressure followed by a sudden fall and death.^3^ A standardized extract from the leaves of *C. odollam* administered intraperitoneally was found to produce a lethal dose in 50% (LD50) of study mice at 20.8 grams per kilogram.^4^ By comparison, the LD50 of purified digoxin in mice is 17.8 milligrams per kilogram when administered orally.^5^

While nonpharmacologic cardiac glycoside toxicity is rare in the United States (U.S.), accounting for 1.5% (677) of the 44,731 plant exposures reported to the National Poison Data System in 2014, *C. odollam* toxicity has demonstrated significant worldwide mortality.^6^ Ritual *C. odollam* poisonings in Madagascar were at one point responsible for the death of 2% of the population.^1^ Poisoning by “pong-pong” seed ingestion is widespread in southern India, with 537 reported deaths in the state of Kerala from 1989–99. Deaths, both homicides and suicides, are almost certainly underreported, as the only method of identifying *C. odollam* toxin is by thin-layer chromatography.^7^ For this reason we were unable to obtain a glycoside blood level; however, we believe that the patient’s history (admitted suicide attempt using “pong-pong” seeds) and hospital course (bradycardia, ECG changes, hyperkalemia) are sufficient to give a diagnosis of *C. odollam* poisoning.

Management of cerberin toxicity is similar to that of digoxin toxicity, consisting of supportive treatments for bradycardia and hyperkalemia as well as administration of digoxin immune fab.^1^,^2^,^8^, ^9^ A Cochrane review stated that digoxin immune fab may be effective for the treatment of yellow oleander poisoning, another poisonous cardenolide-containing plant of the Apocynaceae family.^10^ In 2014 a patient in the U.S. who ingested an unknown number of “pong-pong” seeds was successfully treated with a total of 20 vials of digoxin immune fab.^7^ Narendranathas et al. analyzed 38 sequential *C. odollam* poisonings of which all four patients who presented with normal sinus rhythm survived and nine of the 25 patients with abnormal rhythm on admission died.^11^ More severe poisoning was associated with hyperkalemia, bradycardia (heart rate below 50 bpm), and lack of response to atropine. Our patient was hyperkalemic (5.7 mEq/L) and developed bradycardia, indicating a severe poisoning.

## CONCLUSION

This was the second known *C. odollam* poisoning in the U.S. and the first fatality reported.^7^ Our patient expired despite being treated with the same amount of digoxin immune fab, possibly indicating a more severe poisoning, or reduced affinity between digoxin immune fab and the digoxin-like toxins that our patient ingested. While poisoning from *C. odollam* is rare in Western medicine, clinicians should be aware of its potential morbidity and mortality and be prepared for resuscitative interventions including digoxin immune fab administration, correction of serum potassium concentration, lipid emulsion therapy with consideration of early transfer to a center with extracorporeal membrane oxygenation capabilities.

Documented patient informed consent and/or Institutional Review Board approval has been obtained and filed for publication of this case report.
